# Digital health in Directly Observed Treatment of people with Tuberculosis in the state of São Paulo: Methodological notes

**DOI:** 10.1590/0034-7167.202578supl201

**Published:** 2025-04-25

**Authors:** André Luiz Teixeira Vinci, Nathalia Zini, Willie Otávio Bueno Bernardi, Natacha Martins Ribeiro, Isabela Santana Oliveira, Yan Mathias Alves, Reginaldo Bazon Vaz Tavares, Ione Carvalho Pinto, Ricardo Alexandre Arcêncio

**Affiliations:** I Universidade de São Paulo, Escola de Enfermagem de Ribeirão Preto. Ribeirão Preto, São Paulo, Brazil

Tuberculosis (TB) is a global public health issue, primarily affecting developing countries such as Brazil. According to the World Health Organization (WHO), Brazil is one of the 30 countries with the highest TB burden, ranking 19th, with an incidence of 37 cases per 100,000 inhabitants in 2023 and a mortality rate of 2.72 deaths per 100,000 inhabitants in 2022 ^(1,2)^.

A challenging issue in Brazil is the TB treatment interruption, having national estimates indicating the proportion of 13.6% in 2022, representing a widespread and complex problem. Added to this issue are the catastrophic costs, which affect approximately 50% of individuals with drug-susceptible TB, and when considering the issue of drug-resistant TB, the impact is even higher, with 75% of individuals being economically affected, resulting in the need for new treatment approaches there are more person-centered and manageable for them.

The country is a signatory to the goal of eliminating TB as a public health problem worldwide by 2030 (< 1 case per 100,000 inhabitants), using the “End TB” Strategy as the basis for achieving this goal. This strategy is based on three fundamental pillars: person-centered care, enforced by universal systems; social protection policies; and research and innovation. Thus, the country must seek best solutions based on research and innovation evidence. In this sense, digital health presents itself as a technological resource for care that places the person at the center of their health-disease and care process, thereby promoting greater equity and accessibility.

The use of digital health has been increasingly encouraged, as it provides greater flexibility for the person with TB and the health services, as well as reducing and/or mitigating catastrophic costs for both the individual and the health system. Remote support in therapeutic follow-up enables organizational arrangements based on the needs, preferences, availability, and resources of the person undergoing treatment and the health services, allowing greater co-participation and autonomy in the care process.

In 2022, WHO emphasized the importance of incorporating these resources into Directly Observed Treatment (DOT)^(3,4)^, but their implementation remains more frequent in high-income countries or in specific localities, through isolated initiatives that are carried out sparsely^(3,4)^. Following this recommendation, in 2023 the Brazilian Ministry of Health recommended the incorporation of digital technologies into DOT^(5)^, citing the potential technologies to be used.

Thus, the study “Interaction and effect of the COVID-19 pandemic on tuberculosis control in the state of São Paulo: political-social, clinical-epidemiological aspects and innovative practices” (EPIDOT-TB), which is being developed at the Ribeirão Preto School of Nursing at the University of São Paulo (EERP/USP), aims to evaluate the use of technological innovations in TB treatment supervision. One of its aims is to implement asynchronous video-based DOT supervision, or Video Directly Observed Therapy (VDOT), using an Android smartphone app to record drug intake^(3,6)^. To this end, an open-masked clinical trial with three arms will be conducted, namely: Self-Administered Treatment (SAT); conventional DOT; and VDOT. As it is an interventional study, the project was registered in the Brazilian Clinical Trials System (REBEC - Registration U1111-1310-6499), in addition to approval by the Research Ethics Committee (CAEE: 77580424.5.0000.5393 / Report: 6.945.067). The chosen scenario was the state of São Paulo because it is one of the states having the highest burden of the disease in Brazil, where TB poses a major challenge, mainly due to the social and economic situation.

It will be possible to evaluate the cost-effectiveness of the VDOT strategy in relation to DOT and SAT, based on the TB treatment outcomes, and to estimate, for each type of supervision, the difference in treatment costs for the person, family and health services. The municipal health staff will be involved in the process of incorporating VDOT into the services routine, and will be trained on the advantages of its use for TB individual and for the service, as well as in system and application management.

Additionally, potential changes in the structure, process, and outcome triad of TB treatment in the involved services, resulting from the implementation of the study, will be assessed from the perspective of health professionals. These professionals will also collaborate on data related to treatment costs for the health service and the usability of the VDOT web portal.

Epidemiological data from the Brazilian Information System for Notifiable Diseases (SINAN) of the 645 municipalities of São Paulo state, from 2015 to 2022, were used to define the study settings. The evaluation criteria to identify priority localities were: the TB incidence rate (above 100 cases in the period), the percentage of treatment interruptions (> 10%), and DOT coverage (> 20%). After meeting these criteria, 30 municipalities were selected and invitations were sent out, including all the guidelines regarding the possibility of using VDOT technology in the treatment of people diagnosed with TB.

The current phase of the study implementation includes seven municipalities that accepted the invitation and joined the project to incorporate VDOT technology, with others in the process of contracting or having declined, as shown in the map (Figure 1).

**Figure 1:**
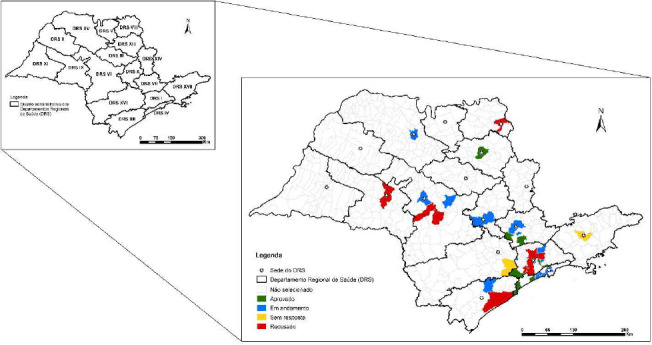
Distribution of municipalities eligible to take part in the project, according to Regional Health Departments, São Paulo, 2024

The VDOT implementation in São Paulo state is expected to offer a sustainable alternative for person-centered treatment follow-up, improving adherence and favorable outcomes, promoting the optimization of resources and costs within the Brazilian universal healthcare system (SUS). The application of this approach in multiple municipalities will also provide a unique view of the monitoring and supervision of the entire treatment process in different health system settings. Thus, the introduction and dissemination of this approach seeks to expand the use of digital health in SUS, advancing toward TB elimination through the use and application of digital technology.

## References

[1] Ministério da Saúde (2024). Boletim Epidemiológico de Tuberculose 2024.

[2] World Health Organization (WHO) (2023). Global tuberculosis report 2023.

[3] World Health Organization (WHO) (2021). Global Tuberculosis Programme: programmatic innovations to address challenges in tuberculosis prevention and care during the COVID-19 pandemic.

[4] Suyanto S, Mertaniasih NM. (2021). The prevalence of drug resistance tuberculosis and its transmission among treatment failure patients in Dr. Soetomo Hospital, Surabaya, Indonesia. J Clin Tuberc Other Mycobact Dis.

[5] Ministério da Saúde (BR) (2023). Nota Informativa nº 20/2023 - CGTM/DATHI/SVSA/MS.

[6] Santos LRA, Linardo ACM. (2020). VDOT - Sistema de Telemonitoramento de Pessoas Acometidas por Tuberculose, 2020. INPI - BR 51.

