# Supplementary material for: Digital health in Directly Observed Treatment of people with Tuberculosis in the state of São Paulo: Methodological notes
Source: Rev Bras Enferm. 2025 Apr 25;78(Suppl 2):e78supl201. doi: 10.1590/0034-7167.202578supl201 (PMC12037184; doi:10.1590/0034-7167.202578supl201)
Supplement: Translation

# Translation: Saúde digital no Tratamento Diretamente Observado da pessoa com Tuberculose no estado de São Paulo: Notas metodológicas

**DOI:** 10.1590/0034-7167.202578supl201pt

**Authors:** André Luiz Teixeira Vinci, Nathalia Zini, Willie Otávio Bueno Bernardi, Natacha Martins Ribeiro, Isabela Santana Oliveira, Yan Mathias Alves, Reginaldo Bazon Vaz Tavares, Ione Carvalho Pinto, Ricardo Alexandre Arcêncio

**Affiliations:** I Universidade de São Paulo, Escola de Enfermagem de Ribeirão Preto. Ribeirão Preto, São Paulo, Brasil

A tuberculose (TB) consiste em um problema de saúde pública em escala global, afetando principalmente países em desenvolvimento, como o Brasil. Segundo a Organização Mundial da Saúde (OMS), o Brasil é um dos 30 países com a mais alta carga da doença, ocupando a 19ª posição, com incidência de 37 casos por 100 mil habitantes em 2023 e mortalidade de 2,72 óbitos para cada 100.000 habitantes em 2022^(1,2)^.

Uma questão desafiadora no Brasil refere-se à interrupção do tratamento de TB, que segundo estimativas nacionais, em 2022, a proporção foi de 13,6%, o que consiste num complexo e amplo desafio^(1)^. Acresce-se a essa questão os custos catastróficos, em que aproximadamente 50% das pessoas com TB sensível têm sido impactadas, e quando considerada a questão da TB resistente, o impacto é ainda mais elevado, 75% das pessoas são afetadas economicamente^(2)^, faz-se necessário novas abordagens para o tratamento, mais centradas na pessoa e suportáveis por elas.

O país é signatário da meta de eliminação da TB como um problema de saúde pública mundial até 2030 (< 1 caso para cada 100.000 habitantes), tendo a Estratégia “*End TB”* como base para o cumprimento dessa meta. A referida estratégia está alicerçada em três pilares fundamentais: cuidado centrado na pessoa, impingido por sistemas universais; políticas de proteção social e a pesquisa e inovação. Assim, cumpre ao país buscar as melhores soluções sustentadas em bases de evidências de pesquisa e inovação. E, nesse sentido, a saúde digital apresenta-se como recurso tecnológico para um cuidado que coloque a pessoa na centralidade do seu processo saúde-doença e cuidado, e, assim, propiciando maior equidade e acessibilidade.

O uso da saúde digital vem sendo cada vez mais incentivado, pois proporciona maior flexibilidade à pessoa com TB e aos serviços de saúde, além da redução e ou mitigação dos custos catastróficos para a pessoa e sistema de saúde. O suporte remoto no acompanhamento terapêutico possibilita arranjos organizacionais mediante as necessidades, preferências, disponibilidades e recursos da pessoa em tratamento e dos serviços, conferindo uma maior coparticipação e autonomia no processo de cuidar.

Em 2022, a OMS ressaltou a importância da incorporação desses recursos na realização do Tratamento Diretamente Observado (TDO)^(3,4)^, mas a sua implantação ainda é mais frequente em países de alta renda ou em localidades específicas, por meio de iniciativas isoladas e realizadas de forma escassa^(3,4)^. Seguindo esta recomendação, o Ministério da Saúde do Brasil recomendou em 2023 a incorporação de tecnologias digitais no TDO^(5)^, citando as possíveis tecnologias para uso.

Assim, o estudo “Interação e efeito da pandemia por COVID-19 no controle da tuberculose no estado de São Paulo: aspectos político-sociais, clínico-epidemiológicos e práticas inovadoras” (EPIDOT-TB), em andamento na Escola de Enfermagem de Ribeirão Preto da Universidade de São Paulo (EERP/USP), tem como objetivo avaliar o uso de inovações tecnológicas para a supervisão do tratamento da TB. Um dos seus objetivos é implantar a supervisão do TDO por meio de vídeos de forma assíncrona, ou *Video Directly Observed Therapy* (VDOT), utilizando um aplicativo para *smartphones* Android para a gravação da ingestão medicamentosa^(3,6)^. Para tal, será realizado ensaio clínico de mascaramento aberto com três braços, a saber: Autoadministrado (TA); TDO convencional e o VDOT. Por tratar-se de um estudo interventivo, o projeto teve registro no Sistema Brasileiro de Pesquisas Clínicas (REBEC - Registro U1111-1310-6499), além da aprovação em Comitê de Ética em Pesquisa (CAEE: 77580424.5.0000.5393/ Parecer: 6.945.067). O cenário escolhido foi o estado de São Paulo por se constituir um dos estados com mais alta carga da doença no Brasil, e onde a TB se faz um grande desafio, principalmente em decorrência da situação social e econômica.

Será possível avaliar o custo-efetividade da estratégia VDOT em relação ao TDO e ao TA, a partir dos desfechos do tratamento de pessoas com TB e estimar a diferença, para cada tipo de supervisão, dos custos de tratamento para pessoa, família e serviços de saúde. As equipes de saúde dos municípios serão envolvidas no processo de incorporação do VDOT na rotina dos serviços, e serão capacitadas sobre os benefícios do seu uso para a pessoa com TB e para o serviço, bem como na realização do manejo do sistema e aplicativo.

Ademais, serão avaliadas possíveis alterações na tríade estrutura, processo e resultado do acompanhamento do tratamento da TB nas unidades envolvidas e resultantes da implementação do estudo, a partir da perspectiva dos profissionais de saúde. Estes também irão colaborar com dados relacionados aos custos do tratamento para o serviço de saúde e o nível de usabilidade do portal web do VDOT.

Para definição dos cenários do estudo, foram utilizados dados epidemiológicos do Sistema de Informação de Agravos de Notificação (SINAN) dos 645 municípios do estado de São Paulo, no período de 2015 a 2022. Os critérios avaliativos para identificar os municípios prioritários foram: a taxa de incidência da TB (acima de 100 casos no período), o percentual de interrupção do tratamento (> 10%) e a cobertura de estratégia de TDO (> 20%). Após cumpridos esses critérios, 30 municípios foram selecionados e procedeu-se o envio de convites, com todas as orientações a respeito da possibilidade de inserção da tecnologia VDOT no tratamento de pessoas diagnosticadas com TB.

A fase atual de implementação da investigação conta com sete municípios que responderam positivamente ao convite e aderiram ao projeto de inserção da tecnologia VDOT, e outros em tramitação de contratualização ou recusaram, conforme apresentado no mapa (Figura 1).

**Figura 1:** Localização dos municípios elegíveis para participação no projeto, segundo Departamentos Regionais de Saúde, São Paulo, 2024

Espera-se que a implantação do VDOT no estado de São Paulo ofereça uma alternativa sustentável para o acompanhamento do tratamento centrado na pessoa, melhorando a adesão e os desfechos favoráveis, promovendo a otimização de recursos e custos dentro do sistema universal de saúde (SUS). O cenário de aplicação em múltiplos municípios também irá proporcionar uma visão única do acompanhamento e supervisão de todo o processo de tratamento em diferentes organizações de sistema de saúde. Assim, a inserção e disseminação dessa abordagem busca ampliar o uso da saúde digital no SUS, possibilitando um avanço rumo à eliminação da TB, com uso e aplicação da tecnologia digital.

# Translation: Salud Digital en el Tratamiento Directamente Observado de Personas con Tuberculosis en el Estado de São Paulo: Notas Metodológicas

**DOI:** 10.1590/0034-7167.202578supl201es

**Authors:** André Luiz Teixeira Vinci, Nathalia Zini, Willie Otávio Bueno Bernardi, Natacha Martins Ribeiro, Isabela Santana Oliveira, Yan Mathias Alves, Reginaldo Bazon Vaz Tavares, Ione Carvalho Pinto, Ricardo Alexandre Arcêncio

**Affiliations:** I Universidade de São Paulo, Escola de Enfermagem de Ribeirão Preto. Ribeirão Preto, São Paulo, Brasil

La tuberculosis (TB) es un problema de salud pública a escala mundial, que afecta principalmente a países en desarrollo como Brasil. Según la Organización Mundial de la Salud (OMS), Brasil es uno de los 30 países con mayor carga de la enfermedad, ocupando el 19º lugar, con una incidencia de 37 casos por 100.000 habitantes en 2023 y una tasa de mortalidad de 2,72 muertes por 100.000 habitantes en 2022^(1,2)^.

Un tema desafiante en Brasil se relaciona con la interrupción del tratamiento de la TB, que según estimaciones nacionales, en 2022, la proporción era de 13,6%, lo que constituye un reto complejo y de gran envergadura^(1)^. A esto se suman los costes catastróficos, en los que aproximadamente el 50% de las personas con TB susceptible se han visto impactadas, y cuando se considera la cuestión de la TB resistente, el impacto es aún mayor, el 75% de las personas se ven afectadas económicamente^(2)^, haciendo necesarios nuevos enfoques de tratamiento más centrados en la persona y soportables para ella.

El país es signatario del objetivo de eliminar la TB como problema de salud pública mundial para 2030 (< 1 caso por cada 100.000 habitantes), con la Estrategia “*End TB”* como base para alcanzar este objetivo. Esta estrategia se basa en tres pilares fundamentales: atención centrada en la persona, aplicada por sistemas universales; políticas de protección social; e investigación e innovación. Corresponde, por tanto, al país buscar las mejores soluciones basadas en las evidencias de la investigación y la innovación. Y en este sentido, la salud digital se presenta como un recurso tecnológico para el cuidado que sitúa a la persona en el centro de su proceso de salud-enfermedad y cuidado, proporcionando así mayor equidad y accesibilidad.

Cada vez se fomenta más el uso de la salud digital, ya que proporciona mayor flexibilidad a la persona con TB y a los servicios sanitarios, además de reducir y/o mitigar costes catastróficos para la persona y el sistema sanitario. El apoyo remoto en el seguimiento terapéutico permite organizarlo en función de las necesidades, preferencias, disponibilidad y recursos de la persona en tratamiento y de los servicios, proporcionando una mayor coparticipación y autonomía en el proceso asistencial.

En 2022, la OMS enfatizó la importancia de incorporar estos recursos al Tratamiento Directamente Observado (TDO)^(3,4)^, pero su implementación aún es más común en países de altos ingresos o en localidades específicas, a través de iniciativas aisladas que se llevan a cabo escasamente^(3,4)^. Siguiendo esta recomendación, en 2023 el Ministerio de Salud de Brasil recomendó la incorporación de tecnologías digitales en el TDO^(5)^, citando posibles tecnologías para su uso.

Así, el estudio “Interacción y efecto de la pandemia de COVID-19 en el control de la tuberculosis en el estado de São Paulo: aspectos político-sociales, clínico-epidemiológicos y prácticas innovadoras” (EPIDOT-TB), en curso en la Escuela de Enfermería de Ribeirão Preto de la Universidad de São Paulo (EERP/USP), pretende evaluar el uso de innovaciones tecnológicas para la supervisión del tratamiento de la TB. Uno de sus objetivos es implementar la supervisión TDO por medio de vídeos asíncronos, o *Video Directly Observed Therapy* (VDOT), utilizando una aplicación de *smartphone* Android para registrar la ingesta de medicamentos^(3,6)^. Para ello, se llevará a cabo un ensayo clínico abierto y enmascarado con tres brazos, a saber: Tratamiento Autoadministrado (TA); TDO convencional y VDOT. Por tratarse de un estudio intervencionista, el proyecto fue registrado en el Sistema Brasileño de Investigación Clínica (REBEC - Registro U1111-1310-6499) y aprobado por el Comité de Ética en Investigación (CAEE: 77580424.5.0000.5393/ Dictamen: 6.945.067). El escenario elegido fue el estado de São Paulo por ser uno de los estados con mayor carga de la enfermedad en Brasil, y donde la TB es un gran desafío, principalmente debido a la situación social y económica.

Será posible evaluar la rentabilidad de la estrategia VDOT en relación con la TDO y la TA, a partir de los resultados del tratamiento de las personas con TB y estimar la diferencia, para cada tipo de supervisión, en los costes del tratamiento para la persona, la familia y los servicios de salud. Los equipos de salud de los municipios estarán involucrados en el proceso de incorporación de la VDOT en la rutina de los servicios, y serán formados en los beneficios de su uso para la persona con TB y para el servicio, así como en la forma de gestionar el sistema y la aplicación.

Además, se evaluarán, desde la perspectiva de los profesionales sanitarios, los posibles cambios en la tríada estructura, proceso y resultado del seguimiento del tratamiento de la tuberculosis en las unidades implicadas y derivados de la aplicación del estudio. También se colaborará con datos relacionados con los costes del tratamiento para el servicio sanitario y el nivel de usabilidad del portal web VDOT.

Para definir los escenarios de estudio se utilizaron datos epidemiológicos del Sistema de Información de Enfermedades de Declaración Obligatoria (SINAN) de los 645 municipios del estado de São Paulo entre 2015 y 2022. Los criterios de evaluación utilizados para identificar los municipios prioritarios fueron: la tasa de incidencia de TB (más de 100 casos en el período), el porcentaje de interrupciones del tratamiento (> 10%) y la cobertura de la estrategia TDO (> 20%). Una vez cumplidos estos criterios, se seleccionaron 30 municipios y enviaron invitaciones con todas las directrices sobre la posibilidad de utilizar la tecnología VDOT en el tratamiento de las personas diagnosticadas de TB.

En la fase actual de aplicación de la investigación hay siete municipios que han respondido positivamente a la invitación y se han sumado al proyecto de inserción tecnológica de VDOT, y otros que están en proceso de contractualización o se han negado, como muestra el mapa (Figura 1).

**Figura 1:** Localización de los municipios elegibles para participar en el proyecto, según los Departamentos Regionales de Salud, São Paulo, 2024.

Se espera que la implementación de VDOT en el estado de São Paulo ofrezca una alternativa sostenible para el seguimiento del tratamiento centrado en la persona, mejorando la adherencia y los resultados favorables, promoviendo la optimización de recursos y costes dentro del sistema sanitario universal (SUS). El escenario de aplicación en múltiples municipios también proporcionará una visión única del seguimiento y supervisión de todo el proceso de tratamiento en diferentes organizaciones del sistema de salud. Así, la inserción y difusión de este enfoque busca ampliar el uso de la salud digital en el SUS, permitiendo avanzar hacia la eliminación de la TB, con el uso y aplicación de la tecnología digital.
